# New York State’s WAVE Method - Evaluation of a Method for Water Quality Monitoring by Citizen Scientists using Benthic Macroinvertebrates

**DOI:** 10.1007/s00267-022-01753-1

**Published:** 2022-12-05

**Authors:** Alene M. Onion, Alexander J. Smith, Brian T. Duffy

**Affiliations:** New York State Department of Environmental Conservation, 625 Broadway, Albany, NY 12233 USA

**Keywords:** Citizen Science, Biological assessment metrics, Benthic macroinvertebrates, Water quality, Natural resource management

## Abstract

New York State Department of Environmental Conservation (NYSDEC) has developed a robust citizen science macroinvertebrate sampling method. The metric relies on the presence and not the absence of key macroinvertebrates and therefore is resistant to collection and sorting errors. It identifies unimpaired streams with high confidence (0.1% type 1 errors) and at a reasonable efficiency compared to NYSDEC’s multimetric index of biological integrity (54%). We rank remaining stream samples for further investigation using a calculated probability of impairment. This method is valuable as a tool for large monitoring programs with limited resources for quality assurance checks. The value of this method goes beyond data collection, however, as data of known quality is an effective communication tool between citizen scientists and state regulatory agencies and/or local decision makers.

## Introduction

Benthic macroinvertebrates are well documented biological indicators in rivers and streams because they are ubiquitous, abundant, diverse, and sensitive to chemical and physical environmental impacts (Barbour et al. 1999; Doughty 1994; Rosenberg and Resh 1993; Roux et al. 1993). Their life cycles are longer and more sedentary compared to other aquatic communities so they are able to detect intermittent discharges, variable concentrations, multiple pollutants, and even those that are not detectable by direct sampling (Rosenberg and Resh 1993). Furthermore, sampling methods are simple and inexpensive compared to water chemistry sampling (Wiedemann 2006). For these reasons, macroinvertebrate sampling is the favored method for most citizen scientist programs focused on stream ecosystems. For example, the states of Arkansas, Colorado, Connecticut, Georgia, Indiana, Kentucky, Maryland, Missouri, New York, North Carolina, Ohio, Oregon, Pennsylvania, and Virginia all have established citizen science programs monitoring water quality using benthic macroinvertebrate communities (Herron et al. 2022).

One disadvantage of benthic macroinvertebrate sampling is that the method is vulnerable to human sources of variability during sample collection, sub-sample sorting, taxonomic identification and enumeration, and calculating assessment metrics (Nerbonne and Vondracek 2003). Some programs have developed approaches to minimize and quantify this uncertainty in data collection and subsequent results. For example, field audits are used to document the level of variability in sample collection to qualify data produced by citizen groups (Boward et al. 2011; Riggert et al. 2017; Virginia Citizen Water Quality Monitoring Program 2007). Other programs limit the role of the citizen scientist to sample collection and sorting and staff perform taxonomic identification and enumeration (Bellucci 2015; Boward et al. 2011). A third and novel approach from Connecticut, uses a presence only macroinvertebrate metric – meaning a metric that considers the presence and not the absence of key macroinvertebrate taxa. A stream sample containing a threshold number of intolerant organisms indicates the stream is fully supporting Connecticut’s aquatic life designated use and any quantity below that threshold produces no conclusion (Bellucci 2015). The presence only metric reduces Type 2 error and vulnerability to human error because it doesn’t presume missing taxa are also missing in the stream system.

The advantage of the presence only metric is that it requires very little time on the part of agency program staff to ensure data quality compared to field audits and limiting the role of citizen scientists. Programs can include more participants while still producing data with high confidence. Citizen scientists can collect data for their own local purposes with very little delay necessary for training and quality assurance. In this way, presence only metrics can increase the likelihood that citizen science programs will benefit environmental management both at the state and local levels.

In this study, we explored the relative value of the presence only metric approach for detecting water quality conditions in New York State. We assessed both a metric that uses the presence of threshold indicator taxa (PTIT) similar to Connecticut’s approach and a metric that uses threshold probabilities of impairment (TPI) based on the presence of these taxa. The New York State Department of Environmental Conservation (NYSDEC) used these results to design the Water Assessments by Volunteer Evaluators (WAVE) program. Our primary objective was to create a volunteer monitoring program that produced data of known quality to enable clear communication of stream health in support of state and local efforts to protect New York’s water resources.

## Methods

To develop both metrics, we used the historic NYSDEC macroinvertebrate data set to (1) identify indicator macroinvertebrates, (2) generate distribution frequencies for each indicator organism / water quality condition combination, and (3) used these indicators and frequencies to calculate and assess the two metrics. To prevent bias, we separated the historic data set into a training data set and a test data set. Steps 1 and 2 were accomplished using the training data set and step 3 was performed on the test data set.

### Working Data Set

The NYSDEC collects water quality data and information in streams and rivers on a statewide, 5‐year cycle. This includes benthic macroinvertebrate samples to estimate water quality impacts on aquatic life. For this project, the focus was on wadeable streams and rivers sampled using a traveling kick method which consisted of a one‐time, 5‐m diagonal transect sample through a riffle area over 5 min. Samplers kick the bottom substrate and collect the dislodged organisms in a 0.25 m X 0.5 m, 900 µm mesh net held downstream. Sampling occurs during the July to September index period. From each sample, a random 100-organism subsample is removed and each individual specimen is identified to the lowest possible taxonomic resolution, typically genus or species (Bode and Novak 1995; NYSDEC 2021; Riva-Murray et al. 2002; Smith et al. 2013, 2007).

We compiled all kick samples from 1990 to 2018. To prevent bias introduced by frequently resampled sites, we restricted the dataset to the most recent sample from each location. This resulted in 2842 samples collected from 1990 unique streams and rivers. We divided this data set randomly into a training data set and a test data set, each with 1421 samples.

### Condition Categories

NYSDEC uses a multimetric index of biological integrity, called the Biological Assessment Profile (BAP) score, to summarize benthic macroinvertebrate data and report water quality impacts on aquatic life. For the traveling kick method, individual component metrics of the BAP include species richness, Hilsenhoff’s biotic index (Hilsenhoff 1988), Ephemeroptera– Plecoptera–Trichoptera richness (Lenat 1988), percent model affinity (Novak and Bode 1992), and the Nutrient Biotic Index—Phosphorus (Smith et al. 2007). BAP scores are calculated by normalizing component metrics to a 10 scale and taking the average. The BAP score is assigned to a four-tiered system of impact category: non (7.5–10), slight (5.0–7.5), moderate (2.5–5.0), or severe (0–2.5) impacts (NYSDEC 2021). A final BAP score below 5 is associated with significant loss of biodiversity, functional organization, and ability to support a balanced community as compared to natural conditions (Karr 1991; Davis and Simon 1995) and suggests that the sampled stream is biologically impaired. A BAP score above 5 indicates that aquatic life in the sampled stream is unimpaired and reflects that of natural conditions or only slightly altered from natural.

### Identifying Indicator Taxa

We used the training data set to identify indicators of impaired and unimpaired biological conditions. Taxonomic resolution was reduced to family level (with five exceptions), reflecting the extent a volunteer would likely be able to visually distinguish taxonomic differences in the field. This decision was based on our experience identifying organisms in the field without a microscope as well as interaction with a broad range of volunteers. Exceptions included Pelecypoda, Hirudinea, and Turbellaria which were reduced to class and Amphipoda to order because of difficulty distinguishing these organisms in the field. We kept *Chironomus* spp. (Diptera: Chironomidae) at genus because they are generally identified by their red coloration.

We selected the indicator taxa from the training data set by comparing taxa present in each condition category. To improve our resolution between impaired and unimpaired categories, we only used non-impacted samples (BAP > 7.5, *n* = 406) to identify indicators of unimpaired biological condition. All moderately and severely impacted (*n* = 287) macroinvertebrate community samples (BAP < 5.0) were used to represent the impaired condition due to the limited number of severely impacted samples (BAP < = 2.5). We calculated the Sørensen index to estimate community similarity within each category and calculated the relative contribution of each taxon. The Sørensen index is the most commonly used index in community ecology to compare populations using presence/absence data (Chao et al. 2006). Selected indicator taxa were more abundant within respective condition categories and contributed less than 2% to the Sørensen index of the opposing category.

### Calculating Distribution Frequencies

We also used the training data set to calculate the frequency of each indicator taxa in each impact category, a necessary step to calculate the probability of impairment. We calculated these frequencies for non and slight impact categories separately but combined the moderate and severe impact categories into one impaired category because the sample sizes were so small. Specifically, frequency was the number of samples containing the indicator taxa divided by the total number of samples in the impaired, slightly impacted, or non-impacted categories. We also calculated the frequency of impaired, slightly impacted, and non-impacted populations overall.

### Calculating and Assessing Metrics

We assessed five possible metrics using the presence of threshold indicator taxa (PTIT) with the test data set. We calculated the frequency at which 3, 4, 5, 6, and 7 minimum indicator taxa correctly or incorrectly (type 1 errors) identified samples collected from unimpaired streams (BAP > 5) or samples from impaired streams (BAP ≤ 5).

We assessed five possible metrics using the TPI also with the test data set. For each sample in the test data set, we calculated the probability of biological impairment and the probability biological condition is unimpaired using a modified Naïve Bayes equation (Eqs. 1 and 2, respectively) (Russell and Norvig 1995). Using the frequencies calculated from the test data set, we calculated the probability a sample in the test data set indicated biological impairment as the frequency of impaired samples multiplied by the frequency each indicator taxa found in the sample is found in impaired samples and divided by the frequency of impaired, slightly impacted, or non-impacted samples overall (Eq. 1). We calculated the probability a sample in the test data set indicated unimpaired conditions using the same equation but with non-impacted frequencies in the numerator (Eq. 2). Finally, we calculated the frequency at which samples with 50%, 70%, 90%, 95%, and 98% minimum probabilities matched the correct or incorrect (type 1 errors) condition category.

1 $$\begin{array}{l}{{{\mathrm{Probability}}}}\,{{{\mathrm{of}}}}\,{{{\mathrm{biological}}}}\,{{{\mathrm{impairment}}}} = \frac{{{{{\mathrm{P}}}}\left( {{{{\mathrm{IM}}}}} \right){{{\mathrm{P}}}}\left( {{{{\mathrm{IT}}}}_{{{\mathrm{1}}}}\left| {{{{\mathrm{IM}}}}} \right.} \right){{{\mathrm{P}}}}\left( {{{{\mathrm{IT}}}}_{{{\mathrm{2}}}}\left| {{{{\mathrm{IM}}}}} \right.} \right) \ldots {{{\mathrm{etc}}}}}}{{{{\mathrm{P}}}}\left( {{{{\mathrm{NI}}}}} \right){{{\mathrm{P}}}}\left( {{{{\mathrm{IT}}}}_{{{\mathrm{1}}}}\left| {{{{\mathrm{NI}}}}} \right.} \right){{{\mathrm{P}}}}\left( {{{{\mathrm{IT}}}}_{{{\mathrm{2}}}}\left| {{{{\mathrm{NI}}}}} \right.} \right) \ldots {{{\mathrm{etc}}}} + {{{\mathrm{P}}}}\left( {{{{\mathrm{SL}}}}} \right){{{\mathrm{P}}}}\left( {{{{\mathrm{IT}}}}_{{{\mathrm{1}}}}\left| {{{{\mathrm{SL}}}}} \right.} \right){{{\mathrm{P}}}}\left( {{{{\mathrm{IT}}}}_{{{\mathrm{2}}}}\left| {{{{\mathrm{SL}}}}} \right.} \right) \ldots {{{\mathrm{etc}}}} + {{{\mathrm{P}}}}\left( {{{{\mathrm{IM}}}}} \right){{{\mathrm{P}}}}\left( {{{{\mathrm{IT}}}}_{{{\mathrm{1}}}}\left| {{{{\mathrm{IM}}}}} \right.} \right){{{\mathrm{P}}}}\left( {{{{\mathrm{IT}}}}_{{{\mathrm{2}}}}\left| {{{{\mathrm{IM}}}}} \right.} \right) \ldots {{{\mathrm{etc}}}}}\end{array}$$

2 $$\begin{array}{l}{{{\mathrm{Probability}}}}\,{{{\mathrm{biological}}}}\,{{{\mathrm{condition}}}}\,{{{\mathrm{is}}}}\,{{{\mathrm{unimpaired}}}} = \frac{{{{{\mathrm{P}}}}\left( {{{{\mathrm{NI}}}}} \right){{{\mathrm{P}}}}\left( {{{{\mathrm{IT}}}}_{{{\mathrm{1}}}}\left| {{{{\mathrm{NI}}}}} \right.} \right){{{\mathrm{P}}}}\left( {{{{\mathrm{IT}}}}_{{{\mathrm{2}}}}\left| {{{{\mathrm{NI}}}}} \right.} \right) \ldots {{{\mathrm{etc}}}}}}{{{{\mathrm{P}}}}\left( {{{{\mathrm{NI}}}}} \right){{{\mathrm{P}}}}\left( {{{{\mathrm{IT}}}}_{{{\mathrm{1}}}}\left| {{{{\mathrm{NI}}}}} \right.} \right){{{\mathrm{P}}}}\left( {{{{\mathrm{IT}}}}_{{{\mathrm{2}}}}\left| {{{{\mathrm{NI}}}}} \right.} \right) \ldots {{{\mathrm{etc}}}} + {{{\mathrm{P}}}}\left( {{{{\mathrm{SL}}}}} \right){{{\mathrm{P}}}}\left( {{{{\mathrm{IT}}}}_{{{\mathrm{1}}}}\left| {{{{\mathrm{SL}}}}} \right.} \right){{{\mathrm{P}}}}\left( {{{{\mathrm{IT}}}}_{{{\mathrm{2}}}}\left| {{{{\mathrm{SL}}}}} \right.} \right) \ldots {{{\mathrm{etc}}}} + {{{\mathrm{P}}}}\left( {{{{\mathrm{IM}}}}} \right){{{\mathrm{P}}}}\left( {{{{\mathrm{IT}}}}_{{{\mathrm{1}}}}\left| {{{{\mathrm{IM}}}}} \right.} \right){{{\mathrm{P}}}}\left( {{{{\mathrm{IT}}}}_{{{\mathrm{2}}}}\left| {{{{\mathrm{IM}}}}} \right.} \right) \ldots {{{\mathrm{etc}}}}}\end{array}$$

where: NI = non-impacted; SL = slightly impacted; IM = impaired; IT = indicator taxa.

## Results

We identified indicator taxa for both unimpaired and impaired biological condition categories (Table 1a and b). The 32 indicators of unimpaired condition were more frequently found in unimpaired samples (composed of both non and slightly impacted samples). The median frequencies of unimpaired indicator organisms in non and slightly impacted samples were 0.17 (IQR: 0.07–0.34) and 0.09 (IQR: 0.02–0.22) respectively compared to the median frequency in impaired samples which was 0.02 (IQR: 0–0.06). Similarly, the 16 indicators of impaired condition were more frequently found in impaired samples. The median frequency of the impaired indicator organisms in impaired samples was 0.11 (IQR: 0.05–0.26) compared to median frequencies in non and slightly impacted samples which were 0.02 (IQR: 0–0.04) and 0.04 (IQR: 0.02–0.12) respectively.

**Table 1:** Macroinvertebrate indicators of unimpaired (BAP>5) and impaired (BAP≤) biological condition and their frequencies in impaired and unimpaired (including non-impacted and slightly impacted) samples

Indicator organisms	Frequencies in unimpaired samples		Frequencies in impaired samples
	Non-impacted samples	Slightly impacted samples	
a. Macroinvertebrate indicators of unimpaired biological condition (BAP > 5) and their frequency in impaired and unimpaired (including non-impacted and slightly impacted) samples.			
(Order: *Coleoptera*)			
Family: *Psephenidae*	0.47	0.5	0.21
(Order: *Diptera*)			
Family: *Athericidae*	0.35	0.25	0.06
(Order: *Ephemeroptera*)			
Family: *Caenidae*	0.24	0.18	0.07
Family: *Baetiscidae*	0	0.01	0
Family: *Ephemerellidae*	0.63	0.25	0.03
Family: *Ephemeridae*	0.04	0.02	0.01
Family: *Heptageniidae*	0.82	0.52	0.21
Family: *Isonychiidae*	0.55	0.36	0.03
Family: *Leptohyphidae*	0.16	0.13	0.04
Family: *Leptophlebiidae*	0.33	0.14	0.02
Family: *Polymitarcyidae*	0.02	0	0
Family: *Potamanthidae*	0.03	0.01	0
(Order: *Megaloptera*)			
Family: *Corydalidae*	0.31	0.26	0.09
(Order: *Odonata*)			
Family: *Gomphidae*	0.23	0.12	0.01
Family: *Capniidae*	0.03	0.02	0
Family: *Chloroperlidae*	0.18	0.06	0
Family: *Leuctridae*	0.27	0.17	0.05
Family: *Nemouridae*	0.04	0.01	0.01
Family: *Peltoperlidae*	0.07	0.02	0
Family: *Perlidae*	0.73	0.42	0.05
Family: *Perlodidae*	0.14	0.03	0
Family: *Pteronarcidae*	0.13	0.02	0
(Order: *Trichoptera*)			
Family: *Brachycentridae*	0.24	0.08	0
Family: *Glossosomatidae*	0.18	0.06	0.02
Family: *Helicopsychidae*	0.07	0.05	0.01
Family: *Hydroptilidae*	0.15	0.21	0.14
Family: *Lepidostomatidae*	0.17	0.03	0.01
Family: *Odontoceridae*	0.11	0.04	0
Family: *Philopotamidae*	0.8	0.6	0.21
Family: *Polycentropodidae*	0.14	0.09	0.06
Family: *Rhyacophilidae*	0.35	0.16	0.03
Family: *Uenoidae*	0.04	0.02	0
b. Macroinvertebrate indicators of impaired biological condition (BAP ≤ 5) and their frequency in impaired and unimpaired (including non-impacted and slightly impacted) samples			
(Order: *Coleoptera*)			
Family: *Haliplidae*	0	0	0.03
(Subphylum: *Crustacea*)			
Family: *Asellidae*	0.02	0.12	0.4
(Subphylum: *Crustacea*)			
Order: *Amphipoda*	0.06	0.22	0.57
(Order: *Diptera*)			
Genus: *Chironomus*	0	0.01	0.13
Family: *Simuliidae*	0.36	0.4	0.38
Family: *Tabanidae*	0.03	0.04	0.07
(Order: *Hemiptera*)			
Family: *Corixidae*	0	0.01	0.02
(Order: *Megaloptera*)			
Family: *Sialidae*	0.03	0.07	0.05
(Class: *Gastropoda*)			
Family: *Lymnaeidae*	0	0.03	0.06
Family: *Physidae*	0.04	0.07	0.19
Class: *Pelecypoda*	0.11	0.13	0.22
(Order: *Odonata*)			
Family: *Cordulegastridae*	0.01	0.01	0.01
Family: *Coenagrionidae*	0.03	0.04	0.1
Family: *Calopterygidae*	0	0.03	0.04
Subclass: *Hirudinae*	0.01	0.03	0.18
Class: *Turbellaria*	0.14	0.21	0.39

The PTIT metric was successfully able to match both impaired and unimpaired biological condition categories for all five taxa threshold iterations. The percent of samples with matching condition categories decreased when we increased the required number of indicator taxa (Tables 2 and 3). Each additional indicator taxa reduced the matching percentage by an average of 12 and 14% for unimpaired and impaired conditions respectively. The frequency of type 1 errors also decreased when we increased the required number of indicator taxa (Tables 2 and 3). Each additional indicator taxa reduced type 1 errors by more than half for both condition categories. For the unimpaired category, there were only 0.1% type 1 errors when we required 6 unique indicator taxa. This was a negligible frequency corresponding to 2 out of the 1421 samples, and both samples had BAP scores (4.88 and 4.99 BAP scores) very close to the unimpaired threshold BAP score (BAP > 5).

**Table 2:** Frequency at which each metric correctly (matching assessments) and incorrectly (type 1 errors) identifies samples from streams with biological conditions that are unimpaired (BAP > 5)

A. Using a threshold number of unique indicator taxa		
Minimum # of indicator taxa	Matching Assessments (percent)	Type 1 errors (percent)
3	89	3.6
4	79	1.1
5	69	0.5
6	54	0.1
7	42	0.0

B. Using a threshold probability		
Minimum Probability	Matching Assessments (percent)	Type 1 errors (percent)
50	80	2.3
70	70	0.8
90	49	0.4
95	39	0.1
98	28	0

**Table 3:** Frequency at which each metric correctly (matching assessments) and incorrectly (type I errors) identifies samples from streams with a biological impairment (BAP ≤ 5)

A. Using a threshold number of unique indicator taxa		
Minimum # of indicator taxa	Matching Assessments (percent)	Type 1 errors (percent)
3	58	11.0
4	35	4.6
5	16	1.5
6	5	0.5
7	2	0.1

B. Using a threshold probability of impairment		
Minimum Probability	Matching Assessments (percent)	Type 1 errors (percent)
50	54	3.2
70	44	1.8
90	26	0.7
95	17	0.3
98	8	0.1

The TPI metric was successfully able to match impaired and unimpaired biological condition categories for all five probability threshold iterations (Tables 2 and 3). As with the PTIT metric, the percent of samples with matching condition categories decreased when we increased the thresholds and the frequency of type 1 errors improved (Tables 2 and 3). This change did not occur at the same rate, however. The PTIT metric matched more unimpaired samples at comparable type 1 error rates than the TPI metric. In impaired samples, the opposite was true with the TPI metric performing better than the PTIT metric.

**Fig. 1:**
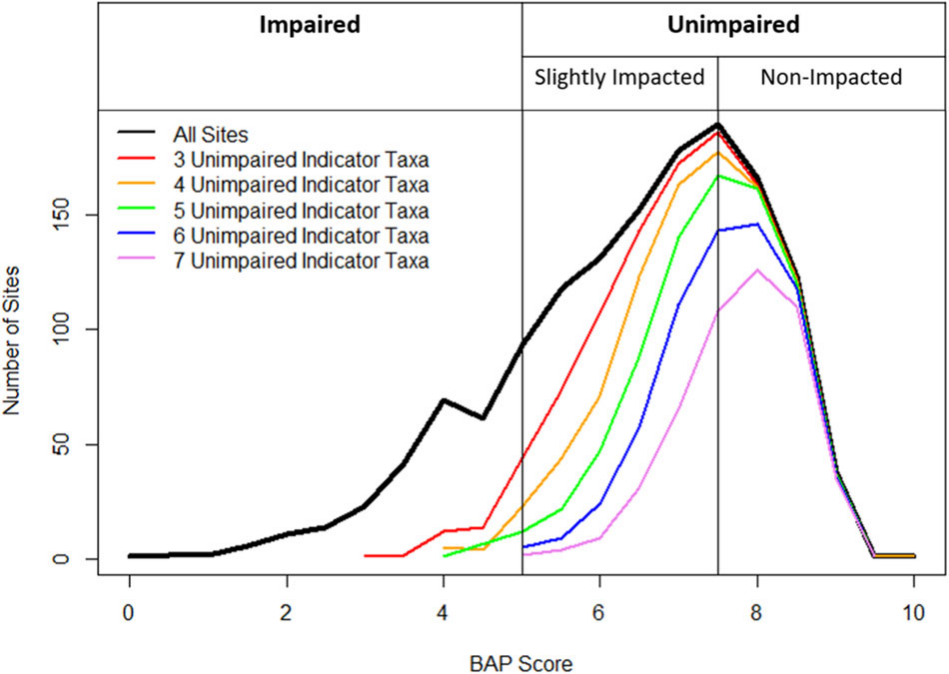
The distribution of all sampling sites (black line) by their defined biological assessment profile scores (BAP) compared to the distribution of those sites with at least one sample containing 3–7 unimpaired indicator taxa (colored lines). The established Impaired (BAP < 5) and unimpaired (BAP ≥ 5) thresholds are delineated as are the more refined slightly impacted (5 ≤ BAP < 7.5) and non-impacted (BAP ≥ 7.5) thresholds

Both metrics performed better at capturing unimpaired compared to impaired biological conditions (Fig. 1, Tables 2 and 3). Both metrics correctly identified more unimpaired samples with fewer type 1 errors compared to impaired samples. For example, PTIT and TPI metrics with a type 1 error rates of 0.1% correctly identified 54% and 39% (respectively) of the unimpaired samples but only 2% and 8% (respectively) of the impaired samples.

## Discussion

Macroinvertebrate sampling is a common method used by citizen science programs sampling rivers and streams (Herron et al. 2022). The data produced can have variable quality because the sampling is vulnerable to human sources of variability (Nerbonne and Vondracek 2003). The presence only metrics developed in this investigation reduce this variability because they rely on the presence rather than the absence of macroinvertebrate taxa. Samples that don’t contain indicator organisms at the required thresholds produce no conclusions and samples that do meet thresholds produce condition assessments with high confidence.

Unimpaired stream samples were identified with high confidence and efficiency by both the PTIT and TPI metrics. We selected the PTIT metric and specifically the threshold of 6 or more indicator taxa for the NYSDEC WAVE Program. This metric identified the greatest number of unimpaired samples (54%) with negligible type 1 errors (0.1%, Table 2) compared to the TPI metric or other iterations of the PTIT metric. The 6 or more taxa threshold is also simpler for external partners to understand than the probability calculations required for the TPI metrics.

The 6 or more PTIT metric selected for the WAVE program is more efficient at identifying high quality stream segments. The metric identified 54% of all unimpaired samples but captured a larger percentage of non-impacted compared to slightly impacted stream segments (Fig. 1). Therefore, studies designed to identify non-impacted waters will be more likely to produce conclusions with the WAVE method than studies in slightly impacted waters.

Impaired stream samples were identified with high confidence but not efficiently by both the PTIT and TPI metrics. Efficiencies were too low (2% and 8% respectively) at negligible type 1 error rates (0.1%) to be valuable as a monitoring tool alone (Table 2). This is likely because abundance, which is a significant driver of genus/species level metrics in impaired waters (Barbour et al. 1999), is deliberately excluded from these presence only metrics. Also, previous research has shown that family level metrics indicate less pollution in stressed streams compared to genus/species level metrics (Hilsenhoff 1988; O’Leary et al. 2004; Penrose and Call 1995). Instead of using this method directly, we use the TPI metric to prioritize WAVE locations for follow up investigations. The probability of impairment is on a continuous scale and therefore is an ideal tool for ranking sites for potential follow up investigation by NYSDEC and application of the BAP.

The NYSDEC launched the WAVE program in 2012 and the program has collected 1343 samples in total, 33% of which indicated unimpaired biological conditions and 7.5% of which launched further investigations of possible impairment. The frequency of unimpaired samples is lower than the 54% in the test data set and is most likely caused by sampling and handling errors that result in no conclusion but, importantly, not erroneous results. WAVE plays an efficient regulatory role because it requires minimal staff time, and the method is robust to most errors. NYSDEC included WAVE sample results in Clean Water Act section 305b reporting.

It is possible to increase the efficiency of WAVE assessments by directing samplers to undisturbed watersheds since the method is more efficient in less disturbed streams (Fig. 1). Connecticut’s Riffle Bioassessment by Volunteers Program began directing participants to less developed, unassessed watersheds and was able to increase the frequency of assessments from 30% to 53% (Lally 2019). This is valuable, especially when a primary goal is to reduce the number of unassessed waters in the state. In New York State, however, we also hope to engage citizen scientists in local protection or restoration efforts. This is particularly important in our state which protects the autonomy of local governments under Article IX “Home Rule” more so than other states (Clark and Cohen 2016). In this way, New York and Connecticut use the same tool differently to accomplish our respective goals.

Citizen scientists transition from data collection to participation in restoration and preservation efforts at multiple levels. With state monitoring programs, citizen scientists can highlight waterbodies that they feel deserve attention and participate in watershed planning efforts supporting those waterbodies (McKinley et al. 2017). At the local level, sampling results generated by citizen scientists can serve to communicate restoration or preservation needs to local town planning boards, watershed groups and/or environmental councils (Fischer 2000).

Initial surveys conducted 2012–2017 indicated that at least 25% of WAVE participants sample to highlight waterbodies of local interest and/or concern to the state monitoring program. Volunteers from two major watersheds participated in state watershed planning efforts. At the local level, watershed organizations and county level monitoring programs use WAVE data to work directly with community leaders to make informed decisions regarding development, tracking of illicit discharges, and natural resource preservation. For example, a watershed group collected samples upstream and downstream of a planned development and presented these results to the town planning board to successfully argue for the stream’s protection (Mike Jastremski, personal communication, January 2015). Several organizations use WAVE as part of a larger education/stewardship program and appreciate that the data can serve both educational and state monitoring objectives. Most WAVE participants, however, indicate that they are sampling primarily to support NYSDEC efforts. Therefore, it is important that we are transparent about the value and application of the data collected to best take advantage of this opportunity to foster stewardship and educate the participants about stream ecosystems and the NYSDEC efforts to conserve and protect these resources. After receiving his results, one WAVE participant wrote, “My family has lived on the banks of Fish Creek since 1866. Helping to assess its health is very rewarding for me” (P. Miller, personal communication, January 5, 2016).

Previous research highlights that citizen science has the potential to improve environmental decision making, but few studies indicate that the data collected changed management decisions (Conrad and Hilchey 2011; Gray et al. 2017). We feel that the primary reason the WAVE program has been effective is because the method produces data of known quality and therefore the data can be accurately used as a tool for communication at multiple levels.

## Conclusions

Presence only metrics are valuable tools for large volunteer programs because they are robust to sampling variability and therefore programs require fewer quality assurance checks to ensure the integrity of the data produced. The NYSDEC WAVE program uses a presence only metric that is not vulnerable to collection and sorting variability thereby removing the major sources of uncertainty. This metric identifies samples from unimpaired streams and we rank the remaining samples for potential follow up investigations using a calculated probability of impairment based on presence only macroinvertebrate types. The benefits of this method go beyond data collection alone. The data is of known quality and thresholds for response are clearly defined which enables clear communication between citizen monitoring groups and NYSDEC and/or local decision makers.
